# Longitudinal Multimodal Assessment in Severe Pneumonia: A Narrative Review of Chest Computed Tomography, Electrical Impedance Tomography, and Digital Lung Sounds

**DOI:** 10.3390/jcm15218623

**Published:** 2026-11-05

**Authors:** Meihong Xia, Xingyue Su, Jing Liu, Di Lv, Jianning Guo, Qin Hui, Fang Ye, Yuanmei Chen, Chao Wang, Lijuan Tang, Qi Zhang

**Affiliations:** 1 Department of Pediatrics, China-Japan Friendship Hospital, Beijing 100029, China; 2 Institute of Clinical Medical Sciences, China-Japan Friendship Hospital, Beijing 100029, China; 3 Chinese Academy of Medical Sciences & Peking Union Medical College, Beijing 100730, China

**Keywords:** severe pneumonia, chest computed tomography, lung ultrasound, electrical impedance tomography, digital lung sounds, longitudinal assessment

## Abstract

Severe pneumonia requires repeated assessment, but anatomical abnormalities, oxygenation, and regional ventilation may follow different recovery trajectories. This narrative review examines chest computed tomography (CT), electrical impedance tomography (EIT), and digital lung sounds as complementary sources of structural, ventilation, and acoustic information, with lung ultrasound (LUS) considered as an important bedside comparator. Focused searches and source verification were conducted in October 2026, with an evidence publication cutoff of 15 July 2026. CT characterizes whole-lung anatomy and complications when clinically indicated; EIT tracks regional ventilation-related changes; and digital auscultation provides stored recordings for serial and remote review. LUS supports bedside assessment of accessible peripheral lesions, pleural abnormalities, and reaeration. Small pneumonia studies support the measurement of regional ventilation trends, and selected controlled studies report favorable short-term findings. Acoustic evidence is concentrated in pediatric populations, with limited adult severe-pneumonia validation. Findings from ARDS and ventilator-weaning studies provide additional physiological context. The proposed longitudinal framework brings these complementary signals together across early assessment, treatment response, ICU transitions, and recovery. Prospective studies should establish its reproducibility, incremental clinical value, resource requirements, and effects on patient-important outcomes before routine implementation.

## 1. Introduction

Severe pneumonia can complicate community-acquired pneumonia (CAP), hospital-acquired pneumonia (HAP), or ventilator-associated pneumonia (VAP), and may be accompanied by acute respiratory failure, sepsis, shock, and other organ dysfunction. These categories differ in pathogen exposure, comorbidity, respiratory support, and prognosis; evidence from one should not automatically be generalized to another [1,2,3,4,5].

Clinical management integrates symptoms and signs, vital signs, oxygenation, microbiology, inflammatory markers, organ function, and imaging. These findings guide antimicrobial treatment, respiratory support, monitoring intensity, and transfer to critical care [1,2,3,4,5]. Treatment trials can establish patient benefit directly: for example, early hydrocortisone reduced 28-day mortality in a defined ICU population with severe CAP [6]. Monitoring studies address complementary questions about measurement, prognosis, and the effect of added information on care.

Longitudinal assessment must distinguish ongoing deterioration, incomplete physiological recovery, and slowly resolving imaging abnormalities. These questions arise during emergency assessment, ward treatment, transitions to or from the ICU, and recovery after discharge. Measurements obtained at different times or under different respiratory-support conditions can be difficult to compare. Additional regional information may help explain discordance and support a more coherent interpretation of the patient’s trajectory.

CT characterizes whole-lung anatomy, EIT measures relative regional impedance changes associated with ventilation, and digital lung sounds preserve respiratory acoustic signals for review. Their different physical principles provide a physiological rationale for a complementary structural–ventilation–acoustic framework, with regional trends interpreted alongside established clinical assessment.

Lung ultrasound (LUS) is an important modality for repeated bedside assessment, including when CT is available. It evaluates accessible peripheral consolidation, pleural abnormalities, dynamic air bronchograms, and semiquantitative aeration patterns [2,7,8,9]. Within this broader toolkit, the CT–EIT–digital-sound focus of this review addresses how complementary signals can inform longitudinal interpretation. LUS is considered throughout as a bedside modality and a relevant comparator when assessing the additional information provided by EIT and acoustic monitoring.

## 2. Literature Sources, Selection, and Scope

This focused narrative review uses a qualitative synthesis of clinical studies, physiological validation studies, guidelines, and relevant reviews. Targeted searches were performed on 15 October 2026 and supplemented by focused searches and source verification on 23 October 2026 using PubMed/MEDLINE-indexed records retrieved through Europe PMC, indexed PubMed abstracts, DOI metadata, and accessible full texts. The evidence publication cutoff was 15 July 2026.

The targeted searches combined pneumonia with electrical impedance tomography; lung ultrasound with pneumonia and monitoring, reaeration, or serial assessment; and digital auscultation, lung sounds, or wireless stethoscopes with pneumonia. A further EIT search addressed randomized studies and ventilator weaning, and a combined-modality query sought pneumonia reports including EIT and acoustic measurements. The queries covered publications from 2000 through 15 July 2026; earlier foundational clinical work was retained when relevant. Reference lists and the existing bibliography also informed selection. Supplementary Material S1 provides exact queries, dates, retrieval counts, selection principles, and source-access limitations.

MX and XS led the literature assessment. Selection was purposive and based on relevance to diagnosis, repeated measurement, physiological validation, clinical decisions, or outcomes. Guidelines and systematic reviews provided context; primary clinical studies were prioritized for Table 1 when design, population, reported participant counts, principal findings, and limitations could be assessed from the available sources. Adult severe-pneumonia studies were distinguished from non-severe or unselected adult CAP, pediatric pneumonia, ARDS, and mixed ventilator-weaning cohorts. Null and discordant findings were retained, including absent correlations with clinical infection scores, outcome-neutral trials, imperfect listener agreement, and reduced performance in unseen participants. Publications from overlapping PERCH cohorts were considered together. The synthesis was qualitative, without formal risk-of-bias scoring or pooled effect estimation.

## 3. The Longitudinal Assessment Gap in Conventional Evaluation of Severe Pneumonia

Conventional assessment combines repeated global measurements with targeted imaging and microbiology. Structural recovery and regional ventilation describe different aspects of the clinical course. Additional regional measurements may help interpret discrepancies among symptoms, oxygenation, imaging, and the response to treatment.

Conventional assessment seeks to determine the source of infection and risk of specific pathogens, define disease extent and complications, identify patients who require intensified monitoring, and guide antimicrobial regimens and the level of nursing care. Severity assessment for CAP should integrate age, comorbidities, mental status, blood pressure, respiratory rate, renal function, oxygenation, and multilobar involvement. Assessment of HAP should additionally consider length of hospitalization, recent antimicrobial exposure, risk of drug-resistant pathogens, and local antimicrobial susceptibility patterns [1,2,3,4,5].

Longitudinal management requires more nuanced clinical judgments because available evidence suggests that imaging abnormalities, oxygenation, inflammation, and regional ventilation recover at different rates. In the classic cohort study by Halm et al., the time to stabilization varied among individual vital signs in patients hospitalized with pneumonia. Median stabilization times were approximately 2 days for heart rate and systolic blood pressure and approximately 3 days for respiratory rate, oxygen saturation, and body temperature. Median time to overall clinical stability ranged from 3 days under lenient criteria to 7 days under strict criteria and increased with disease severity [10]. Radiographic recovery often lags behind improvement in symptoms and vital signs; studies of severe CAP suggest that residual short-term chest radiographic abnormalities do not necessarily indicate uncontrolled infection [11]. The causes of post-discharge return visits or readmissions are also highly heterogeneous. Secondary infection, extrapulmonary complications, aspiration, comorbidity burden, and functional decline may all contribute. Systematic reviews further suggest that chronic respiratory disease, heart failure, chronic kidney disease, diabetes, cancer, and post-discharge follow-up arrangements are associated with readmission risk [12,13,14].

Individual measures have different limitations. Inflammatory markers describe systemic responses rather than regional ventilation, and their trajectories may be discordant with clinical or imaging recovery. SpO_2_ and blood gases describe global gas exchange and must be interpreted with oxygen delivery, circulatory status, respiratory effort, and sampling conditions. CT provides anatomical detail but involves discrete acquisitions, radiation, transport, and resource use. These constraints do not apply equally to all imaging: LUS can be repeated at the bedside without ionizing radiation [8,9]. A combined study should therefore compare its added information with realistic serial usual care, including LUS where available.

A useful longitudinal signal must be reproducible under defined conditions, add information to existing measures, and ultimately improve a decision or an outcome. These are separate evidential requirements.

The evidence summarized in Table 1 supports different levels of inference. Diagnostic studies show additional CT findings; physiological studies describe EIT or LUS changes during treatment; and acoustic studies assess recording feasibility or classification performance. The complete CT–EIT–digital-sound strategy remains a proposed framework: no comparative clinical study identified in this focused review established its outcome benefit. This distinction between measurement validity, predictive association, and clinical utility informs the interpretation of the individual modalities and their potential combination.

**Table 1:** Selected primary clinical studies: populations, findings, and limits of applicability to longitudinal assessment in adults with severe pneumonia.

Study/Modality/Design	Population and Sample	Principal Findings	Limitations and Applicability
Claessens 2015 [15] CT; prospective diagnostic-impact study	319 ED adults with suspected CAP	CT changed diagnostic probability in 187/319 (58.6%); 80% of changes agreed with day-28 adjudication.	Adults with suspected CAP of varying severity; non-randomized diagnostic-impact study with management changes assessed after CT.
Upchurch 2018 [16] CT; multicenter prospective surveillance	2251 hospitalized adults with CAP; 66 CT-only cases	CT-only and radiograph-positive cases had similar ICU admission (23% vs. 21%) and ventilation rates (6% vs. 5%).	Selectively ordered CT; diagnostic comparison in hospitalized adults, with ICU admission and ventilation as observed outcomes.
Karhu 2016 [17] CT; observational paired-image review	65 selected ICU adults with severe CAP from 457 patients	CT disclosed 38 additional findings in 34/65; 26/34 underwent an intervention/procedure.	Highly selected CT-imaged subset of a severe-CAP ICU cohort; observational paired-image assessment.
Bouhemad 2010 [18] LUS; serial physiological study	30 critically ill patients with VAP; day 0 and day 7	LUS reaeration change correlated with CT reaeration (rho = 0.85).	Small single-center ventilated VAP cohort; the endpoint was imaging-based reaeration during antibiotic treatment.
Chiumello 2018 [19] LUS; crossover physiological study	20 sedated, paralyzed ARDS adults; PEEP 5 and 15 cm H_2_O	LUS reflected CT aeration; score changes did not track PEEP-induced recruitment (R = 0.01).	Indirect ARDS evidence under controlled ventilation and specified PEEP levels; aeration and recruitment represent different physiological responses.
Pradhan 2020 [20] LUS; single-center diagnostic RCT	200 intubated patients randomized (98 LUS, 102 control); 88 VAP diagnoses (44/group)	Ventilator-free days 7.80 vs. 3.7 (*p* = 0.044); ICU mortality/stay, ventilator days, and antibiotic duration not significantly different.	LUS plus clinical assessment vs. CXR plus clinical assessment; single-center, open-label diagnostic-strategy trial.
Karsten 2014 [21] EIT; serial observational study	24 CAP patients; admission and day 5–6	Ventilation distribution corresponded with radiographic abnormalities and became more homogeneous.	Small uncontrolled CAP cohort; severity mix incompletely described in the accessible report.
Jiang 2021 [22] EIT; prospective observational study	35 patients with non-severe pneumonia; before treatment and day 6	GI and CoV improved, but their changes did not correlate with CPIS change.	Non-severe pneumonia population; uncontrolled before–after measurements, with discordance between EIT and CPIS changes.
Zhao 2025 [23] EIT-guided airway clearance; prospective controlled study	50 mechanically ventilated adults aged ≥60 years with severe pneumonia; two ICUs; 25/group	Reported extubation success 88% vs. 56% (*p* = 0.0255), with favorable short-term physiological findings.	Reported as randomized but also labeled observational; small sample, short follow-up, and differing therapy intensity; study-specific protocol rules.
Xiong 2026 [24] EIT response measurement in a randomized BAL trial	60 intubated very elderly patients with severe pneumonia; BAL vs. no bronchoscopy	Weaning success 60.0% vs. 33.3% (*p* = 0.038); regional EIT changes favored BAL; ICU mortality not significantly different.	Randomization compared BAL with no bronchoscopy; EIT measured the regional response in a small, selected intubated population.
He 2021 [25] EIT-guided PEEP; open-label RCT	126 ARDS adults randomized; 117 analyzed after 9 exclusions	No significant difference in 28-day mortality (21% vs. 27%), ventilator-free days, or ICU stay.	Indirect ARDS evidence; single center, 24-h intervention, post-randomization exclusions; limited power.
Yuan 2026 [26] EITVent; five-center randomized trial	190 adults with moderate-to-severe ARDS; EIT-guided PEEP vs. lower PEEP/FiO_2_ table	28-day mortality 55.9% vs. 52.6%; reported HR 0.96 (95% CI 0.65–1.41); ventilator-free days not significantly different.	Stopped early for futility, limiting precision; the favorable higher-recruitability subgroup signal was exploratory within an overall neutral ARDS trial.
Wisse 2024 [27] EIT; observational SBT study	23 analyzed ventilated adults; 18 SBT successes/5 failures	GI differed by SBT result; EELI changes did not. EIT did not distinguish subsequent extubation success/failure.	Mixed diagnoses; 3 subsequent extubation failures; movement/contact artifacts; the accessible abstract reports 24 participants.
Zhang 2026 [28] EIT-derived flow index (EFI); multicenter observational SBT cohort	150 ventilated adults; 107 passed SBT and were extubated; 7 reintubations within 48 h	SBT outcome AUC 0.980; reintubations occurred below the same-cohort EFI threshold of 1.333.	Same-cohort threshold derivation and exploratory reintubation analysis with only 7 events; mixed ventilated population.
Murphy 2004 [29] Digital sounds; score development and prospective diagnostic test	Development: 50 pneumonia/50 controls; test: 50/50; total 200	Test sensitivity 0.78 and specificity 0.88 at study-selected score > 6.	Single hospital with asymptomatic controls; diagnostic-score testing in a selected population.
McCollum 2017 [30] PERCH; recording/reliability substudy	792 case and 301 control recordings; children 1–59 months	89.5% of case recordings interpretable; agreement for any abnormality 74.9% (PABAK 0.50).	Pediatric syndromic pneumonia; noise/crying and moderate agreement; overlaps later PERCH analyses.
McCollum 2020 [31] PERCH; observational association analysis	792 recorded cases; 742 interpretable; radiographic subset 491; outcome subset 618	No acoustic category independently predicted radiographic pneumonia among children with danger signs.	Pediatric cohort; missing data, an imperfect reference standard, and overlapping participants; observational acoustic associations.
Park 2022 [32] PERCH; method-agreement analysis	383 pediatric cases with paired assessment within 2 h; expanded analyses in 737	Digital/conventional agreement: PABAK 0.50 for any crackles/wheeze; 0.30 for crackles-only; 0.27 for wheeze.	Different classification patterns; conventional assessment not a definitive gold standard; overlapping PERCH cohort.
Kaimakamis 2024 [33] Digital sounds; prospective observational study	173 intubated COVID-19 ARDS adults; 579 sessions/3393 recordings	Selected acoustic features associated with respiratory mechanics and outcomes.	Single-center, exploratory associations, no independent predictive validation; controlled ventilation and pathogen-specific setting.

## 4. Structural Assessment: Chest CT and Bedside Lung Ultrasound

When clinically indicated, CT delineates the distribution and extent of consolidation, ground-glass opacity, atelectasis, pleural disease, necrosis, and abscess, and can help evaluate alternative diagnoses [15,16,17,34,35]. In 319 emergency-department patients with suspected CAP, CT changed the estimated diagnostic probability in 58.6% [15]. CT-only pneumonia in a hospitalized adult CAP cohort had pathogen and outcome profiles resembling radiograph-positive disease [16]. In a selected series of 65 ICU patients with severe CAP who had paired imaging, CT disclosed additional findings in 34 patients [17]. These studies illustrate its value for diagnostic clarification and the assessment of complications.

CT is not suitable for continuous monitoring. Intrahospital transport to the CT suite carries a risk of adverse events for critically ill patients and those requiring continuous oxygen or respiratory support. Before imaging, the expected diagnostic benefit should therefore be weighed against transport safety [36,37,38]. Requirements for CT equipment, maintenance, infrastructure, and specialist staffing also limit accessibility in primary care, remote areas, and resource-limited settings [39]. The effective dose of a conventional adult chest CT is approximately 6–8 mSv, compared with approximately 0.1 mSv for conventional two-view chest radiography; the actual dose varies with the scanner, acquisition protocol, scan range, patient body habitus, and institution [40,41,42,43].

As ultra-low-dose acquisition and artificial intelligence (AI)-based denoising develop, the radiation burden of stage-specific CT reassessment may be reduced further. In a prospective proof-of-concept study of 54 immunocompromised adults, AI-denoised ultra-low-dose chest CT had a median effective dose of 0.12 mSv, approximately 1.95% of that of conventional-dose CT, while maintaining relatively high detection accuracy for pneumonia and some related findings [44]. Interpretation should account for the small sample, the selected immunocompromised population, and the use of conventional-dose CT as the principal reference standard. Broader application requires evaluation in the intended clinical population.

Repeat CT should address a defined clinical question, such as suspected complications, an alternative diagnosis, or an inadequate response that remains unexplained after assessment [1,2,3,4,5,34,35]. Clinically acquired scans can provide the anatomical reference for longitudinal multimodal comparison, with scan timing determined by the patient’s needs and the balance between diagnostic benefit, radiation, and transport burden.

Longitudinal CT interpretation should consider the interval since treatment initiation, lesion distribution, changes in complications, and the accompanying clinical trajectory. Residual opacities can be interpreted alongside oxygenation, symptoms, and regional bedside measurements, particularly when structural resolution appears slower than physiological recovery [11,35,36,37,38,39,40,41,42,43].

LUS provides an independent bedside advantage for repeated assessment. A meta-analysis of 10 studies including 1172 adults reported pooled sensitivity of 94% and specificity of 96% for pneumonia diagnosis, although operator expertise, protocols, and reference standards varied [7]. LUS can document accessible consolidation, air bronchograms, pleural effusion, and aeration patterns without transport or radiation [8,9]. Dynamic air bronchograms and serial changes add physiological context to bedside structural assessment.

Direct longitudinal evidence includes 30 patients with VAP examined before antibiotics and on day 7. Change in a 12-region LUS reaeration score correlated with CT-measured reaeration (Spearman rho = 0.85) [18]. This small single-center study supports measurement of treatment-associated aeration change. Aeration is distinct from recruitability: in 20 ventilated ARDS patients, LUS scores reflected CT aeration, but score changes did not track CT-measured PEEP-induced recruitment (R = 0.01, *p* = 0.67) [19].

A single-center diagnostic randomized trial compared LUS plus clinical findings with chest radiography plus clinical findings for VAP diagnosis [20]. Among 200 randomized intubated patients (98 LUS and 102 control), 88 were diagnosed with VAP, with 44 diagnoses per group. The study reported more ventilator-free days with the LUS strategy (7.80 versus 3.7 days; *p* = 0.044), but no significant differences in ICU mortality, ICU stay, ventilator days, or antibiotic duration. These findings provide decision-impact evidence relevant to the inclusion of LUS in longitudinal assessment.

LUS and CT provide complementary anatomical information. LUS may miss deep abnormalities separated from the pleura by aerated lung, and access, obesity, dressings, scanning settings, and operator training affect reproducibility [8]. CT offers broader coverage and characterization of deep lesions and complications. EIT follows relative regional ventilation-related impedance changes with lower anatomical resolution [45,46,47]. Integration can therefore relate bedside aeration and ventilation trends to the anatomical distribution of disease, while comparative studies should include usual care incorporating LUS where available.

## 5. EIT: Bedside Monitoring of Regional Ventilation Trends

EIT permits repeated or continuous bedside measurement of relative regional impedance changes without ionizing radiation. It provides a means of following regional ventilation trends under documented acquisition conditions and relating them to the clinical course.

A thoracic electrode belt applies low-amplitude alternating currents and records surface electrical potentials. Reconstruction yields images of impedance change associated with regional changes in air content. Common outputs include tidal impedance variation, end-expiratory lung impedance (EELI), center of ventilation (CoV), global inhomogeneity index (GI), and regional ventilation delay. EIT has limited anatomical resolution and samples a thoracic volume around the belt rather than the entire lung. EELI represents relative changes in end-expiratory impedance under specified acquisition conditions, rather than an absolute whole-lung volume [45,46,47].

Consensus statements address acquisition, processing, terminology, and selected ICU applications [45,46,47]. Comparisons with electron-beam CT, quantitative CT, and four-dimensional CT support physiological validity for regional ventilation or air-content changes [48,49,50,51]. In spontaneously breathing adults, interpretation should additionally account for belt position, posture, effort, and respiratory-support changes, which may alter the measured signal.

Direct pneumonia studies are small and heterogeneous. Karsten et al. followed 24 CAP patients, observing more homogeneous ventilation during treatment [21]. A preliminary pediatric CAP study reported agreement with radiography for the affected side, but its age group limits transfer to adult disease [52]. In 35 patients explicitly described as having non-severe pneumonia, Jiang et al. found improvement in GI and CoV after treatment; however, changes in these indices did not correlate with changes in the Clinical Pulmonary Infection Score (CPIS) [22]. Zhao et al. studied 50 mechanically ventilated older adults with severe pneumonia in two ICUs and reported extubation success of 88% with EIT-guided airway clearance versus 56% with fixed-schedule treatment [23]. This positive signal warrants acknowledgment, but the article labels the design both randomized and observational, follow-up was short, and the intervention differed in therapy intensity as well as EIT feedback. A single case report in severe pneumonia with obesity provides only hypothesis-generating evidence [53].

A further randomized trial enrolled 60 intubated very elderly patients with severe pneumonia and compared bronchoscopy with bronchoalveolar lavage against no bronchoscopy [24]. It reported higher weaning success (60.0% versus 33.3%) and favorable regional EIT changes, without a significant ICU mortality difference. Randomization tested bronchoscopy/lavage, while EIT measured the regional response. The study illustrates the use of EIT to characterize treatment-associated physiological changes in a selected population.

Regional ventilation fraction, GI, and CoV, together with within-session EELI responses, can be interpreted alongside signal quality and measurement conditions. In patients with asymmetric disease or discordant clinical findings, paired recordings may help characterize how regional ventilation changes during treatment, positioning, activity, or airway-clearance procedures. Such comparisons provide clinically relevant questions for prospective evaluation and build on the measurement findings of the pneumonia studies [21,22,23,24].

## 6. Digital Lung Sounds: Objective, Trackable, and Remotely Reviewable Acoustic Data

Digital auscultation preserves respiratory acoustic signals for replay, remote interpretation, and algorithmic analysis. Linking recordings to time, anatomical site, and acquisition conditions creates a reviewable record for longitudinal comparison.

Lung sounds are among the most readily obtainable bedside signals. Conventional auscultation can rapidly identify crackles, wheezes, rhonchi, coarse crackles, diminished breath sounds, and abnormal upper-airway sounds [54,55], but it is subjective and does not preserve the examination for later comparison. A meta-analysis of auscultation in adults with acute pulmonary disease reported a pooled sensitivity of approximately 37% (95% confidence interval [CI], 30–47%) and a pooled specificity of approximately 89% (95% CI, 85–92%), with substantial between-study heterogeneity [56]. Thus, an abnormal finding may raise suspicion of disease, whereas normal auscultation does not reliably exclude it. Auscultation should therefore be used as a clinical clue rather than as a basis for definitive diagnosis or exclusion.

Early primary evidence includes Murphy et al.’s acoustic-score development and prospective test study, with 100 participants in each phase. Test sensitivity was 0.78 and specificity 0.88 when pneumonia cases were compared with asymptomatic controls [29]. That selected comparator and single-hospital design limit application to adults with severe pneumonia and competing diagnoses. In the pediatric PERCH studies, digital recordings were feasible, but listener agreement was imperfect [30,31,32]. The 2020 analysis included 792 recorded cases and 742 interpretable recordings, with smaller radiographic and outcome subsets. Among children with WHO danger signs, no acoustic category independently predicted radiographic pneumonia [31]. Digital and conventional classifications also differed, with only fair-to-moderate agreement for several sound categories [32]. These publications include overlapping participants rather than three independent validation cohorts.

Systematic reviews describe substantial heterogeneity in devices, annotation, reference standards, and external validation [57,58,59]. Nocturnal monitoring demonstrates the feasibility of repeated recordings in children with pneumonia [60]. In rural Bangladesh, nine non-physician health workers obtained quality recordings in 867 of 990 children aged 2–59 months (87.6%) after three days of training and supervised practice; 766 of 853 quality recordings with timing data (89.8%) were acquired within five minutes [61]. An AI study in hospitalized Malawian children evaluated agreement with an expert listening panel [62]. In a four-hospital pediatric wireless-stethoscope study, sensitivity and specificity exceeded 92% in subject-dependent experiments, whereas subject-independent performance was markedly lower; the accessible abstract reported values only above 50% for diagnosis and above 39% for prognosis [63]. The contrast highlights the importance of evaluating performance in previously unseen patients.

In adults, a single-center prospective observational study of 173 intubated patients with COVID-19 ARDS found associations between selected acoustic features, respiratory mechanics, and outcomes [33]. Controlled ventilation and severe viral ARDS differ from ward-based or recovering pneumonia. Pediatric findings also require revalidation because chest-wall properties, respiratory rates, sound transmission, airway disease, and etiologies differ by age [30,31,32,57,58,59,60,61,62,63]. Device and site changes, background noise, and the prevalence of alternative pulmonary disease can further alter performance.

Crackles, wheezes, rhonchi, and diminished breath sounds have multiple possible causes and require interpretation in relation to location, clinical findings, and the recording conditions [54,55,56]. Serial recordings can document changes in the distribution and character of abnormal sounds when anatomical sites, device settings, respiratory conditions, and recording quality are recorded consistently. Paired acoustic and EIT observations may then help characterize whether an airway-related acoustic change accompanies a change in regional ventilation.

## 7. Integrated Interpretation of CT, EIT, and Digital Lung Sounds

The modalities differ in spatial coverage and physical basis. CT depicts whole-lung anatomy; LUS examines accessible pleural and peripheral regions; EIT reconstructs relative ventilation-related impedance changes around the belt; and acoustic sensors sample chest-wall sites [8,35,45,46,47,54,55]. An integrated wearable vest recorded EIT and chest sounds in a feasibility study of 20 healthy adults [64]. Anatomical registration is approximate, so integrated interpretation emphasizes the location and direction of longitudinal changes. For example, consolidation on CT, reduced EIT ventilation in a corresponding region, and persistent local crackles or diminished breath sounds can be considered together when assessing whether a structural abnormality remains associated with regional ventilation impairment.

Residual CT opacity accompanied by improving regional EIT ventilation, fewer abnormal sounds, and clinical stability may support the interpretation that structural resolution is lagging behind physiological recovery. If regional ventilation remains reduced despite limited CT abnormalities, acquisition quality, posture, effort, airway obstruction, and pleural disease deserve consideration. Increasing wheeze with greater ventilation inhomogeneity may draw attention to airway narrowing, while fewer rhonchi after airway clearance can be compared with the accompanying EIT trend. These paired observations organize plausible explanations for the clinical course and identify questions for reassessment.

Discordance should prompt review of acquisition quality and the clinical problem. The appropriate imaging modality depends on the suspected complication, stability, and available expertise. LUS can rapidly assess pleural effusion, accessible consolidation, or pneumothorax at the bedside; CT may be needed for deep lesions, necrosis, abscess, or unresolved diagnostic questions [8,9,34,35]. New respiratory distress or instability requires prompt standard evaluation; obtaining multimodal recordings or CT must not delay urgent care.

Table 2 compares the measurements and their principal limits. Together, the modalities offer a way to relate lesion distribution, regional ventilation, and acoustic trends over time. Evaluation of integration should compare the combined information with its individual components and serial usual care, with prespecified handling of missing data and discordant findings.

## 8. Potential Clinical Application Scenarios for Multimodal Assessment

**Figure 1:**
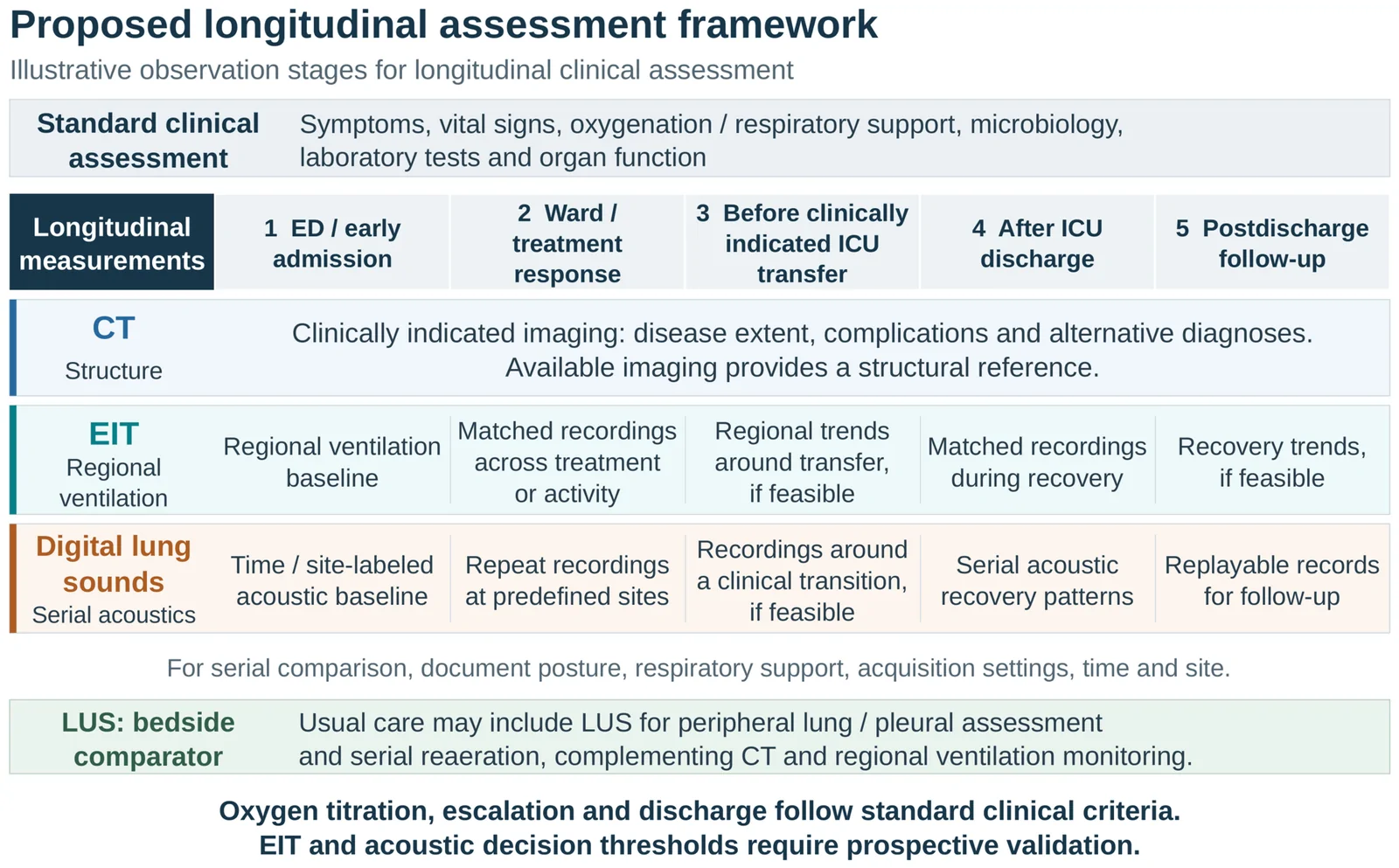
Proposed longitudinal multimodal assessment framework using CT, EIT, and digital lung sounds, with LUS as a complementary bedside modality. Timing is illustrative. CT is performed when clinically indicated, and standard clinical assessment guides care.

Potential applications span emergency assessment, ward treatment, ICU transitions, and recovery after hospital discharge. Clinical assessment, microbiology, gas exchange, and indicated imaging remain the basis of care [1,2,3,4,5]. Within this setting, CT provides an anatomical reference when clinically indicated, EIT characterizes regional ventilation trends, and digital lung sounds provide replayable acoustic observations; LUS contributes repeated bedside structural and aeration assessment. Figure 1 and Table 3 illustrate opportunities to pair these observations across the care pathway. EIT and acoustic thresholds for oxygen titration or level-of-care decisions have not been externally validated in severe pneumonia.

Within-patient comparison requires documentation of posture, respiratory-support modality and settings, oxygen delivery, breathing pattern and effort, belt level and contact, recording sites, device settings, duration, and artifacts [8,45,46,47,55]. FiO_2_ may be uncertain during low-flow oxygen therapy, and unchanged oxygen flow does not guarantee comparable inspired oxygen or ventilation. Necessary respiratory support should never be withheld to obtain a comparable recording. Changes in acquisition conditions should be recorded and incorporated into interpretation. Across repeated sessions, comparisons should prioritize regional tidal-ventilation distribution under matched acquisition conditions, belt position, and analysis definitions. EELI changes are assessed relative to the baseline of a stable continuous recording [46,47].

During emergency assessment or early admission, regional ventilation and acoustic baselines may be obtained after stabilization and paired with contemporaneous clinical data. In patients with multilobar or asymmetric abnormalities, EIT can describe regional ventilation distribution, while recorded crackles, wheeze, or diminished breath sounds provide acoustic context. Available CT or LUS findings help relate these signals to the distribution of disease. Prehospital acquisition requires separate feasibility assessment because movement, limited access, staff workload, and noise differ from hospital conditions.

On the ward, a paired assessment at an illustrative interval of 24–72 h after treatment initiation can address whether changes in symptoms, inflammation, and oxygen requirement are accompanied by changes in regional ventilation and abnormal lung sounds. EIT may show redistribution of ventilation or a change in GI under comparable acquisition and analysis conditions; within-session EELI responses can be assessed during stable continuous recordings. Repeat sound recordings document the location and character of acoustic changes. Comparison with clinically indicated imaging can help interpret a lag between structural and physiological recovery or an unexplained lack of improvement. The observation interval should be selected according to the clinical question and specified prospectively in studies.

Before ICU transfer or other clinically indicated escalation, contemporaneous EIT and acoustic observations may help characterize regional ventilation impairment within the documented episode of deterioration. During oxygen reduction, activity, positioning, or airway clearance already prescribed for clinical reasons, paired recordings can describe the associated regional response. These observations should be considered together with oxygenation, respiratory effort, hemodynamics, mental status, and organ function [1,2,3,4,5], with the clinical indication and the timing of measurements recorded explicitly.

After ICU discharge, the most recent imaging can serve as a structural reference for re-establishing regional ventilation and acoustic baselines. An observation window such as 24–48 h is illustrative. Recordings before and after prescribed mobilization, positioning, or changes in respiratory support can help characterize regional ventilation, breathing-pattern changes, and persistent abnormal sounds during recovery. Interpretation should also include cough effectiveness, swallowing and airway concerns, oxygenation, activity tolerance, and the patient’s clinical rehabilitation plan.

Before and after hospital discharge, standardized recordings offer a potentially useful record of recovery for patients with persistent sputum production, exertional dyspnea, chronic lung disease, or prolonged respiratory-support needs. A pre-discharge acoustic baseline can support subsequent remote review; centers with appropriate resources may also retain an EIT baseline. Symptoms and clinical follow-up provide the context for interpreting later changes. Feasibility, adherence, uninterpretable recordings, false alarms, and access to timely clinical review are relevant endpoints for evaluating these applications. Table 3 summarizes the corresponding observation stages and clinical questions.

## 9. Implications of Evidence from the ICU

ICU studies inform measurement mechanisms and possible intervention targets, but ARDS, controlled ventilation, and prolonged weaning are not interchangeable with adult severe pneumonia. Pneumonia may cause ARDS, yet ARDS cohorts include other etiologies, and ventilator settings directly affect both EIT and acoustic signals.

HFNC studies show that EIT can capture support-associated physiological changes and have explored prediction of treatment failure [65,66]. ARDS studies address individualized PEEP, recruitment and overdistension, and prone-position physiology [25,67,68]. A single-center randomized trial analyzed 117 adults after post-randomization exclusions and found no significant difference in 28-day mortality (21% versus 27%), ventilator-free days, or ICU stay with a 24-h EIT-guided PEEP strategy [25]. The five-center EITVent randomized trial included 190 adults with moderate-to-severe ARDS and was stopped early for futility [26]. Overall 28-day mortality did not differ significantly between EIT-guided PEEP and a lower PEEP/FiO_2_ table (55.9% versus 52.6%; reported hazard ratio 0.96, 95% CI 0.65–1.41), nor did ventilator-free days. Findings in the higher-recruitability subgroup remain exploratory, and early termination limits precision.

Spontaneous-breathing-trial studies provide further information about regional ventilation during weaning [27,69]. In Wisse et al., 23 participants were analyzed in the full-text results: GI differed by trial success, but EELI changes did not, and EIT did not distinguish subsequent extubation success from failure [27]. The single-center VISION study derived a threshold of >20% for the absolute ventral-to-dorsal ventilation difference in 55 ventilated adults and prospectively evaluated this risk threshold for failure of rapid liberation in a later cohort of 43 at the same center [70]. More recently, a prospective multicenter cohort enrolled 150 ventilated adults; 107 passed the trial and were extubated, and seven required reintubation within 48 h [28]. The EIT-derived flow index discriminated trial outcome (AUC 0.980), but the 1.333 threshold came from that same cohort. Its association with reintubation was exploratory, involved few events, and required external validation.

Acoustic work includes an extubation-monitoring pilot and real-ICU algorithm evaluation [71,72]. In the latter, 701 recordings yielded an AUC of 0.75 with raw audio and 0.80 after band-pass filtering; other denoising approaches did not improve performance [71]. The unit of analysis was the recording, and the task was sound classification. Adult COVID-19 studies additionally describe exploratory associations with respiratory mechanics and outcomes [33]. These findings inform technical development and the selection of clinically relevant endpoints for future monitoring studies.

## 10. Practical Considerations for Clinical Implementation and Research Design

Practical use of complementary measurements depends on the clinical question, local expertise, and available resources. Combining devices adds acquisition, synchronization, interpretation, cleaning, maintenance, and staff-training demands. CT requires scanner and transport resources; LUS requires operator skill and chest access; EIT requires dedicated equipment, stable electrode contact, and trained interpretation; digital acoustics adds device, software, data-storage, and review requirements [8,35,36,37,38,39,45,46,47,55]. Resource assessment should include total staff time, consumables, failed recordings, technical support, and downstream investigations as well as purchase price. Local measurement of these costs and workloads is more informative than a universal cost estimate. Table 4 lists minimum reporting items for evaluable studies.

A staged evaluation should first determine acquisition feasibility, inter- and intra-operator repeatability, between-device agreement, and the proportion of usable data. Protocols should prospectively specify target populations, timing relative to treatment, handling of support changes, reference standards, and adverse or burdensome consequences. Subsequent multicenter cohorts should test incremental predictive value against serial usual care, including LUS where available, and compare the combined model with each component. Decision-impact or randomized studies are then needed to determine whether access to the signals changes appropriate care and patient-important outcomes.

Consecutive enrollment and transparent exclusions can reduce spectrum bias. Models should separate patients, rather than sound segments from the same patient, between training and validation; external assessment should include different institutions, devices, age groups, and respiratory-support settings. Calibration, missingness, false alarms, and prespecified thresholds matter alongside discrimination. Cutoffs derived from exploratory samples require prospective validation in the target population. Reporting and appraisal can use TRIPOD+AI, PROBAST+AI, DECIDE-AI, and CONSORT-AI as appropriate to the study design [73,74,75,76].

Future studies should prespecify patient-important endpoints such as treatment failure, need for respiratory support, ICU readmission, functional recovery, and resource use, while recording additional tests, interventions, and effects on the timing of care.

## 11. Conclusions

CT, EIT, and digital lung sounds provide complementary structural, regional ventilation, and acoustic information for longitudinal assessment in severe pneumonia. LUS adds repeated bedside structural and aeration assessment and is an important comparator. Integrating these measurements with established clinical evaluation offers a coherent way to interpret asynchronous recovery and discordant findings across early assessment, treatment response, ICU transitions, and follow-up. The framework provides a basis for prospective evaluation of reproducibility, incremental clinical value, resources, and patient-important outcomes.

## Figures and Tables

**Table 2:** Comparison of CT, LUS, EIT, and digital lung sounds for longitudinal pneumonia assessment.

Modality	Measurement and Coverage	Role Supported by Current Evidence	Principal Limits
CT	Whole-lung anatomy; deep lesions, extent of opacities, and complications	Diagnostic clarification and clinically indicated reassessment [15,16,17,34,35]	Radiation, transport, discrete acquisition; anatomical change does not directly measure tidal ventilation.
LUS	Accessible pleural/peripheral abnormalities, dynamic air bronchograms, and aeration patterns	Bedside diagnosis and repeat aeration assessment [7,8,9,18,19]	Deep lesions may be shielded by aerated lung; access/operator/protocol dependence; aeration change not synonymous with recruitment.
EIT	Relative regional tidal impedance changes and EELI around belt level	Physiological trend measurement under documented conditions [21,22,23,45,46,47,48,49,50,51,52,53]	Limited anatomical coverage/resolution; motion, electrode contact, posture, effort, and respiratory-support changes affect comparability.
Digital lung sounds	Acoustic patterns recorded at defined chest-wall sites	Storable/replayable recordings; selected diagnostic and feasibility evidence [29,30,31,32,33,54,55,56,57,58,59,60,61,62,63]	Nonspecific sounds; noise and device dependence; age/setting shifts and limited adult severe-pneumonia validation.

**Table 3:** Potential observation stages and paired assessments for longitudinal multimodal evaluation; timing is illustrative and guided by the clinical setting.

Stage	CT Task	EIT Focus	Digital Lung Sound Focus	Clinical Question and Supportive Paired Assessment
Emergency department/early after admission	Use clinically indicated CT to characterize disease, alternative diagnoses, and complications; LUS adds bedside assessment	Regions with reduced ventilation, ventilation distribution, and baseline GI	Location and character of crackles, wheezes, and diminished breath sounds	How do regional ventilation and acoustic findings relate to imaging and the initial clinical assessment? Establish paired baselines after stabilization.
24–72 h after treatment initiation (illustrative)	Review available imaging; repeat CT only for an unresolved clinical question	Regional ventilation fraction and GI under comparable acquisition and analysis conditions; within-session EELI responses where continuously recorded	Longitudinal changes in the distribution and character of abnormal sounds	Are clinical improvement and regional ventilation trends concordant? Compare paired recordings with symptoms, oxygenation, and clinically indicated imaging.
Before ICU transfer	Select imaging according to suspected complications and patient stability	Regional ventilation changes and inhomogeneity during deterioration; within-session EELI responses where continuously recorded	New or changing diminished breath sounds, crackles, or wheeze	What regional changes accompany clinically recognized deterioration? Relate available paired observations to gas exchange, respiratory effort, and imaging.
After ICU discharge	Use recent imaging as a structural reference; repeat CT when clinically indicated	Regional trends during prescribed support changes, positioning, and mobilization	Persistence or change in crackles, rhonchi, wheeze, and upper-airway sounds	What changes accompany recovery and prescribed activity? Pair observations with cough effectiveness, oxygenation, and activity tolerance.
Follow-up before/after hospital discharge	Select follow-up imaging according to symptoms, risk, and clinical indication	A pre-discharge regional ventilation baseline and subsequent trends where feasible	A replayable acoustic baseline and standardized recordings for remote review	How does regional and acoustic recovery relate to symptoms and function? Assess feasibility and concordance with established clinical follow-up.

**Table 4:** Minimum reporting information for longitudinal multimodal studies in severe pneumonia.

Item	Minimum Reporting Information
Patients and setting	CAP, HAP, or VAP; explicit severity criteria; age, comorbidity, immune status, respiratory support, recruitment method, exclusions, and care setting.
Conventional assessment and timing	Symptoms, vital signs, SpO_2_/blood gases, oxygen delivery, inflammatory and microbiological findings, severity score, treatment timing, and the clinical reason for escalation/de-escalation.
CT and LUS	Clinical indication and acquisition time; CT protocol/dose and lesion distribution; LUS zones/probe/settings, inaccessible zones, scoring method, stored clips, and reader agreement.
EIT acquisition	Device/software, belt level/contact/reapplication, posture, duration, effort, respiratory-support settings, artifact/missing-data handling, ROI definitions, and prespecified outputs (e.g., EELI, GI, CoV).
Acoustic acquisition	Device, sampling/filtering, anatomical sites, duration, posture/support, noise, uninterpretable recordings, annotation procedures, and patient-level training/validation separation.
Comparators and outcomes	Serial usual care including LUS where available; single-component comparisons; prespecified clinical and physiological outcomes, calibration, external validation, and whether disclosed results changed decisions.
Feasibility, resources, and harms	Setup/recording/review time, staffing/training, consumables and maintenance, usable-data proportion, access/adherence, false alarms, extra tests/interventions, delayed care, and local costs.

## Data Availability

No new data were created or analyzed in this study. Data sharing is not applicable to this article.

## Supplementary Materials

The following supporting information can be downloaded at: https://www.mdpi.com/article/10.3390/jcm15218623/s1. Supplementary Material S1: Targeted Search and Source Verification Record provides exact queries, search dates, retrieval counts, selection principles, and source-access limitations.

## Abbreviations

ARDS	Acute respiratory distress syndrome
AUC	Area under the receiver operating characteristic curve
BAL	Bronchoalveolar lavage
CAP	Community-acquired pneumonia
CI	Confidence interval
CONSORT-AI	Consolidated Standards of Reporting Trials–Artificial Intelligence
COPD	Chronic obstructive pulmonary disease
CoV	Center of ventilation
COVID-19	Coronavirus disease 2019
CPIS	Clinical Pulmonary Infection Score
CXR	Chest radiography
CT	Computed tomography
DECIDE-AI	Developmental and Exploratory Clinical Investigations of DEcision support systems driven by Artificial Intelligence
ED	Emergency department
EELI	End-expiratory lung impedance
EFI	EIT-derived flow index
EIT	Electrical impedance tomography
FiO_2_	Fraction of inspired oxygen
GI	Global inhomogeneity index
HAP	Hospital-acquired pneumonia
HFNC	High-flow nasal cannula
HR	Hazard ratio
ICU	Intensive care unit
LUS	Lung ultrasound
PABAK	Prevalence- and bias-adjusted kappa
PEEP	Positive end-expiratory pressure
PERCH	Pneumonia Etiology Research for Child Health
PROBAST+AI	Prediction model Risk Of Bias ASsessment Tool–Artificial Intelligence
RCT	Randomized controlled trial
ROI	Region of interest
RVD	Regional ventilation delay
SBT	Spontaneous breathing trial
SpO_2_	Peripheral oxygen saturation
TIV	Tidal impedance variation
TRIPOD+AI	Transparent Reporting of a multivariable prediction model for Individual Prognosis Or Diagnosis–Artificial Intelligence
VAP	Ventilator-associated pneumonia
WHO	World Health Organization
