# Supplementary material for: Longitudinal Multimodal Assessment in Severe Pneumonia: A Narrative Review of Chest Computed Tomography, Electrical Impedance Tomography, and Digital Lung Sounds
Source: J Clin Med. 2026 Nov 5;15(21):8623. doi: 10.3390/jcm15218623 (PMC13764463; doi:10.3390/jcm15218623)
Supplement: Supplementary file 1 [file jcm-15-08623-s001.zip › jcm-4644732-supplementary.pdf]

Supplementary Material S1

Targeted Search and Source Verification Record

jcm-4644732 | Searches: 15 and 23 October 2026 | Evidence publication cutoff: 15 July 2026

This record documents focused literature retrieval and source verification for a narrative review. The searches were performed on 15 October 2026 and retained an evidence publication cutoff of 15 July 2026. Supplementary focused searches and source checks were performed on 23 October 2026; the publication cutoff was retained.

Sources were PubMed/MEDLINE-indexed records retrieved through Europe PMC (SRC:MED), DOI metadata, indexed abstracts, and accessible full texts. Metadata were retrieved for all records returned by the five specific queries below: 39, 142, 42, 236, and zero records, respectively. Counts overlap and must not be summed as unique studies. Relevant records and cited studies received focused qualitative review; full texts were not available for every study. The process was a purposive narrative selection without duplicate independent screening, formal risk-of-bias scoring, or pooled analysis.

1. EIT_pneumonia

SRC:MED AND ((TITLE_ABS:"electrical impedance tomography") AND (TITLE_ABS:pneumonia) AND FIRST_PDATE:[2000-01-01 TO 2026-07-15])

Search date: 15 October 2026. Matches returned by service: 39. Records retrieved: 39. Archived JSON: search-EIT_pneumonia.json.

The complete request URL and retrieved records are archived in revision-search-log.json and the JSON file named above.

2. LUS_serial_pneumonia

SRC:MED AND ((TITLE_ABS:"lung ultrasound" OR TITLE_ABS:"ultrasound assessment") AND (TITLE_ABS:pneumonia) AND (TITLE_ABS:monitoring OR TITLE_ABS:reaeration OR TITLE_ABS:serial) AND FIRST_PDATE:[2000-01-01 TO 2026-07-15])

Search date: 15 October 2026. Matches returned by service: 142. Records retrieved: 142. Archived JSON: search-LUS_serial_pneumonia-complete.json.

The complete request URL and retrieved records are archived in revision-search-log.json and the JSON file named above.

3. digital_sounds_pneumonia

SRC:MED AND ((TITLE_ABS:"digital auscultation" OR TITLE_ABS:"lung sound" OR TITLE_ABS:"wireless stethoscope") AND TITLE_ABS:pneumonia AND FIRST_PDATE:[2000-01-01 TO 2026-07-15])

Search date: 15 October 2026. Matches returned by service: 42. Records retrieved: 42. Archived JSON: search-digital_sounds_pneumonia.json.

The complete request URL and retrieved records are archived in revision-search-log.json and the JSON file named above.

4. EIT_outcomes_weaning

SRC:MED AND ((TITLE_ABS:"electrical impedance tomography") AND (TITLE_ABS:randomized OR TITLE_ABS:weaning) AND FIRST_PDATE:[2000-01-01 TO 2026-07-15])

Search date: 15 October 2026. Matches returned by service: 236. Records retrieved: 236. Archived JSON: search-EIT_outcomes_weaning-complete.json.

The complete request URL and retrieved records are archived in revision-search-log.json and the JSON file named above.

5. combined_modalities

SRC:MED AND ((TITLE_ABS:pneumonia) AND (TITLE_ABS:"electrical impedance tomography") AND (TITLE_ABS:"lung sound" OR TITLE_ABS:"digital auscultation" OR TITLE_ABS:"acoustic") AND FIRST_PDATE:[2000-01-01 TO 2026-07-15])

Search date: 15 October 2026. Matches returned by service: 0. Records retrieved: 0. Archived JSON: search-combined_modalities.json.

The complete request URL and retrieved records are archived in revision-search-log.json and the JSON file named above.

Selection and interpretation

MX and XS led the literature assessment. Sources were selected purposively for relevance to diagnosis, serial physiological measurement, measurement reliability, treatment or decision impact, and outcomes. Primary studies informed Table 1; reported designs, participant counts, findings, and source uncertainties are stated from the available sources. Null and conflicting findings were retained. Overlapping PERCH cohorts were considered together, and study-specific thresholds were distinguished from externally validated clinical thresholds.

The 60-patient bronchoalveolar-lavage trial (Xiong 2026; PMID 41795859) provides evidence on regional EIT changes accompanying treatment; its randomization tested lavage. A case ultimately diagnosed as cryptogenic organizing pneumonia and a perioperative study without a baseline severe infectious pneumonia population were outside the direct severe-pneumonia evidence scope.

Other selected studies include the five-center EITVent trial (Yuan 2026; PMID 41738148), which reported an overall null mortality result and early futility termination; the multicenter EFI observational cohort (Zhang 2026; PMID 42219497), whose threshold was derived in the same cohort and whose reintubation analysis involved few events; and the single-center randomized LUS diagnostic-strategy trial in VAP (Pradhan 2020; PMID 32361220), which reported more ventilator-free days but no significant mortality or other reported outcome differences. The publisher's Results preview reports 200 randomized patients (98 LUS, 102 control) and 88 VAP diagnoses (44 per group). The manuscript distinguishes findings supported by indexed abstracts and metadata from information available in full text.

Search counts describe query retrieval, not numbers of screened or included studies or patients. The zero-result combined-modality query is interpreted within the scope of this focused retrieval, alongside reference checking and the existing bibliography.

Supplementary focused searches and source checks — 23 October 2026

These checks addressed paired EIT–sound acquisition, ventilator-liberation thresholds, measurement comparability, and the Pradhan trial denominator. They supplement the five 15 October queries above. The companion file jcm-4644732_supplementary search and source record_20261023.json contains exact queries, dates, request URLs, retrieval counts, and returned record identifiers. Counts overlap and are not inclusion counts. Queries extending beyond 15 July were exploratory checks; no later publication was incorporated into the manuscript.

The 2023 healthy-volunteer integrated-vest study and the 2025 VISION study were added after these focused checks. VISION used 55 derivation and 43 later validation participants at the same center. Its temporal validation is distinguished from the same-cohort EFI threshold and from external validation for severe-pneumonia oxygen or level-of-care decisions. Existing EIT consensus sources [46,47] support within-session EELI analysis and matched repeated-session regional-ventilation comparisons. The existing 2026 Bangladesh feasibility study supplies a pediatric primary-care acquisition example.

Pradhan source: https://www.sciencedirect.com/science/article/abs/pii/S0883944120305463 (Results section preview, accessed 23 October 2026). Its randomized and VAP denominators are reported separately. The trial registration NCT03917888 was checked as a distinct source; its enrollment field is not substituted for the published randomized count.
